# On the Liability, Relatively Considered, of the Adult Teeth to Caries

**Published:** 1855-04

**Authors:** Thomas Underwood

**Affiliations:** Surgeon Dentist.


### ARTICLE III.

### *On the Liability, relatively considered, of the Adult Teeth to Caries.* By Thomas Underwood, Surgeon Dentist.

Calculations, such as the following, are of use to the dental practitioner in enabling him to determine what teeth he should elect to remove in abnormal condition where caries does not exist.

The only tables of a similar kind that I am aware of, are those contained in John Tomes' paper on the same subject, in which the number was 3000; in mine I have restricted myself to 200 consecutive cases, 100 male and 100 female, in order to see how far the analogy will be kept up. The condition in life (a very necessary point to be observed in such calculations) is similar in both cases, John Tomes' being taken from patients at the Middlesex Hospital, mine from those of the Royal Free Hospital, the parties being consequently of the poorer classes. It is worth observing that during the time I removed 100 teeth for women I extracted but 82 for men.

| Of these 200 permanent teeth, | | John Tomes 3000 permanent teeth, | |
|---|---|---|---|
| 2 | were central incisors. | 72 | were central incisors. |
| 6 | lateral incisors. | 117 | lateral incisors. |
| 5 | canines. | 78 | canines. |
| 21 | first cuspids. | 273 | first bicuspids. |
| 28 | second bicuspids. | 434 | second bicuspids. |
| 72 | first molars. | 1124 | first molars. |
| 46 | second molars. | 637 | second molars. |
| 20 | third molars. | 265 | third molars. |
| 200 | | 3000 | |

Let my table be multiplied by 15 to bring it up to John Tomes' number and the result will be

| | | | | |
|---|---|---|---|---|
| 2×15 | 30 central incisors. | 72×15 | 1080 first molars.. |
| 6 | 90 lateral incisors. | 46 | 690 second molars. |
| 5 | 75 canines. | 20 | 300 third molars. |
| 21 | 315 first bicuspids. | | —— |
| 28 | 420 second bicuspids. | | 3000 |

The analogy between the two tables is extremely striking, sufficiently so to enable us to predict with tolerable certainty which teeth in a perfectly sound set will first be affected with caries, the chief discrepancy being in the incisor teeth, which may in a measure be accounted for from the fact, that my cases do not include any removed from insufficient size of the jaw.

Of these 200 cases,

| | Men lost. | Women lost. |
|---|---|---|
| Central incisors, . . | 0 | 2 |
| Lateral incisors, . . . | 3 | 3 |
| Canines, . . . . . | 1 | 4 |
| First bicuspids, . . . | 8 | 13 |
| Second bicuspids, . . | 10 | 18 |
| First molars, . . . | 37 | 35 |
| Second molars, . . . . | 32 | 14 |
| Third molars, . . . | 9 | 11 |

It thus appears that with men the greater tendency to caries is in the molar, with women in the front and bicuspid teeth.

The loss of teeth in men and women on the right, in comparison with the left side, will appear in the following table:

| | Men's upper jaw. | | Men's lower jaw. | |
|---|---|---|---|---|
| | Right side. | Left side. | Right side. | Left side. |
| Central incisors, | 0 | 0 | 0 | 0 |
| Lateral incisors, | 0 | 1 | 1 | 1 |
| Canines, | 0 | 0 | 0 | 1 |
| First bicuspids, | 5 | 1 | 2 | 0 |
| Second bicuspids, | 4 | 5 | 1 | 0 |
| First molars, | 11 | 12 | 7 | 7 |
| Second molars, | 6 | 7 | 11 | 8 |
| Third molars, | 2 | 2 | 4 | 1 |
| | — | — | — | — |
| | 28 | 28 | 26 | 18 |

| | Women's upper jaw. | | Women's lower jaw. | |
|---|---|---|---|---|
| | Right side. | Left side. | Right side. | Left side. |
| Central incisors, | 1 | 1 | 0 | 0 |
| Lateral incisors, | 1 | 0 | 1 | 1 |
| Canines, | 2 | 2 | 0 | 0 |
| First bicuspids, | 5 | 4 | 3 | 1 |
| Second bicuspids, | 5 | 4 | 4 | 5 |
| First molars, | 14 | 7 | 6 | 8 |
| Second molars, | 4 | 1 | 4 | 5 |
| Third molars, | 3 | 1 | 5 | 2 |
| | 35 | 20 | 23 | 22 |

We here perceive that the teeth in the upper are more liable to caries than those in the lower jaw, and also that there is a greater loss on the right than the left side of the mouth, and are naturally anxious to discover the cause of these phenomena. The acknowledged predisposition of the right side of the body to disease, as compared with the left, presents itself to us as a solution of one of our difficulties, but why should the teeth in the upper maxilla be more attacked by caries than those in the lower? May it not be owing to the facts that the latter are more bathed in saliva, more in contact with and consequently more cleansed by the tongue. Confessedly this fact deserves more attention and investigation than has hitherto been conferred upon it, and the attention of the readers of the Journal is earnestly recommended to the subject.

The utility of tables, such as these, is evident in a variety of cases. Take, for instance, those which are constantly presenting themselves to the practitioner where, from an overcrowded state of the jaw it is necessary one of the bicuspids should be removed, the dentist will at once decide that the usually adopted operation of extracting the first bicuspid is inexpedient, the tables showing that the second are more liable to caries, and ought, therefore, to be removed.

At some future period it will afford me much pleasure to contrast these tables with an equal number of cases taken from the higher classes of society, who, from their position and wealth, can obtain for themselves and children a constant attention to their dental organs, I shall now be able to accomplish this in a shorter period, having requested my friend and partner, Dr. James Robinson, to furnish me with the results of his observations on this subject.

